# Elucidating the linkage between non-volatile precursors and particulate-phase flavor compounds in mainstream smoke *via* multi-platform analysis and cascade ultrafiltration

**DOI:** 10.3389/fpls.2026.1868514

**Published:** 2026-07-14

**Authors:** Yi-Han Zhang, Jin Yan, Hai-Tao Wang, Ai-Min He, Xiao-Ru Wang, Wei Shen, Ling-Bo Ji, Bao-Jiang He, Xiu-Juan Xu, Xiao Yang, Min Wang, Ren-Qi Wang, Hao-Ke Zang

**Affiliations:** 1 China Tobacco Hebei Industry Co., Ltd., Shijiazhuang, China; 2 Zhengzhou Tobacco Research Institute of China National Tobacco Corporation, Zhengzhou, China; 3 School of Food and Biological Engineering, Shaanxi University of Science and Technology, Xi’an, China

**Keywords:** flavor, LC-MS, membrane filtration, nontargeted analysis, particulate

## Abstract

**Introduction:**

Understanding the chemical linkage from non-volatile precursors in leaf to flavor compounds in mainstream smoke is critical for evaluating the contrasting sensory perceptions of different tobacco phenotypes.

**Methods:**

To elucidate this mechanism, we systematically compared high-quality Zimbabwe tobacco leaf and China Hehua tobacco dust using a multi-platform analytical strategy, including cascade membrane ultrafiltration, UV-*vis*, ^1^H NMR, gas chromatography-mass spectrometry (GC-MS) and liquid chromatography-high resolution mass spectrometry (LC-HRMS).

**Results:**

The results indicated that the bulk macromolecules were predominantly carbohydrates and highly conserved across both tobacco leaves, while trace chemical divergences drive their distinct sensory profiles. Zimbabwe tobacco smoke particulates were enriched in aromatic pyrazoles and other N-heterocycles, fueled by a nitrogen-rich precursor matrix in the uncombusted extract of Zimbabwe tobacco leaf. Conversely, Hehua smoke featured sweet-toned furans and heavy alkanes, originating from an altered chemical balance alongside a carbon-hydrogen-oxygen (CHO) fraction that is relatively more aliphatic and lipid-rich. Furthermore, by tracking nontargeted markers in the smoke particulates generated from filtrates and retentates attained through the cascade filtration stages, potential macromolecular interactions were found influencing the combustion transformation. Specific macromolecular fractions of the 50 to 300 kDa range in the Hehua matrix exhibit pronounced co-enrichment with free small-molecule precursors.

**Discussion:**

The observed co-enrichment suggests that these specific macromolecular fractions may act as carriers during their transfer into mainstream smoke. This novel comparative framework successfully bridges the analytical gap between uncombusted tobacco precursors and flavors in smoke particulates, establishing a chemical foundation for the digital quality evaluation and targeted formulation of substitute tobacco materials.

## 1 Introduction

The unique geographical location and distinct ecological environment of tobaccos shape their metabolic profiles and sensory characteristics (Jiang et al., 2024; Li et al., 2025; Wu et al., 2026). Zimbabwe occupies a central role in the global tobacco market, ranking as the world’s sixth-largest producer and third-largest exporter of high-quality tobacco (Woelk et al., 2001). Given this significant market presence, there is an increasing need for rigorous scientific analysis of Zimbabwe tobacco ingredients with objective, digital quality evaluation to replace traditional, subjective manual grading (Cole and Cole, 2024). In addition, as an agricultural product, its production is limited by infrastructural and policy factors, prompting many countries to seek alternative tobacco materials to substitute Zimbabwe tobacco when it is in short supply (Lencucha et al., 2020). Comparative studies have been conducted to evaluate the flavor principles distinguishing Zimbabwe tobacco from other regional varieties, such as those from China, Brazil, Canada, and the United States (Xiang et al., 2010; Peng et al., 2024). Nonetheless, tobaccos substituted cannot fully replace genuine Zimbabwe tobacco leaves because they lack the typical tobacco flavor profile, cause discomfort, and exhibit poor combustibility.

Approximately 9, 600 chemical substances have been detected in tobacco leaves and their smoke (Rodgman and Perfetti, 2013). A diverse array of flavor compounds, including polyphenols, indoles, organic acids, alcohols, esters, lactones, and terpenoids, constitutes the complex aroma profile of tobacco leaves (Xu et al., 2025). Importantly, minor flavor components delivered much higher sensory improvements than the mixture built purely from the major flavor components (Yang X. et al., 2025). Because tobacco presents such a complicated chemical matrix, nontargeted analysis is favorable over targeted analysis, allowing comprehensive evaluation of complex mixtures without prior compositional knowledge while identifying unknowns and uncovering complete chemical profiles (Adeniji et al., 2023). Currently, this research is predominantly conducted using gas chromatography-mass spectrometry (GC-MS) and liquid chromatography-high resolution mass spectrometry (LC-HRMS). However, the reported investigations by MS possess inherent limitations. GC-MS focuses primarily on volatile compounds, while LC-HRMS is generally restricted to profiling the small molecules within the uncombusted tobacco leaves rather than the combusted material (Li et al., 2025; Xu et al., 2025; Yang M. et al., 2025; Wu et al., 2026).

Understanding the critical linkage between the precursors in the tobacco leaf and the formed compounds in mainstream smoke is essential because the chemical profile of mainstream smoke directly determines the sensory perception of a combustible tobacco product. Analysis on simulated heating rates and oxygen-depleted environments of a burning cigarette has revealed that most flavor compounds remain unchanged during combustion (Baker et al., 2004). This high transfer rate occurs because these substances rapidly distill and vaporize out of the high-temperature heat zone before they can reach their thermal decomposition thresholds. Once transferred, the majority of the biologically active and sensory-relevant compounds in mainstream smoke reside in the particulate phase, making the analysis of this trapped particulate matter crucial for accurately characterizing the true sensory and flavor profile of the smoke (Borgerding and Klus, 2005).

In addition to MS-based nontargeted analysis investigations, spectroscopic techniques such as UV-*vis* and NMR have emerged as powerful tools for the comprehensive characterization of tobacco. UV-*vis* spectrophotometry, when combined with multivariate chemometric tools like linear discriminant analysis, provides a rapid and effective method for authenticating and discriminating cigarette tobacco blends based on their hydrophilic extracts, achieving near-perfect classification accuracy (Giokas et al., 2011). Meanwhile, NMR spectroscopy offers a non-destructive approach for detailed metabolic and structural profiling. ^1^H NMR metabolomic fingerprinting has proven highly effective at differentiating between wild-type and transgenic tobacco plants by mapping key shifts in primary and secondary metabolites, including sugars, organic acids, and defense-related compounds (Choi et al., 2004). Beyond the uncombusted leaf, NMR has also been successfully applied to directly analyze native mainstream tobacco smoke particulate matter. This approach preserves the native chemical environment, enabling the *in situ* measurement of free-base nicotine fractions and the quantification of complex smoke constituents (Barsanti et al., 2007). While these techniques have proven invaluable for characterizing either the raw leaf or the mainstream smoke independently, integrating them to bridge the chemical gap between uncombusted macromolecular precursors and particulate-phase flavor compounds in mainstream smoke has not yet been fully realized.

To bridge the current analytical gap and uncover the true chemical foundation of tobacco smoke sensory perception, this study investigates the critical linkage between non-volatile macromolecular precursors and the derived flavor compounds in the particulate phase of mainstream smoke. We developed a comprehensive, multi-platform analytical framework to compare a premium Zimbabwe tobacco with a substitute domestic variety (i.e., Hehua tobacco dust), tracing the compositional evolution from liquid extracts to mainstream smoke. Specifically, liquid extracts of both tobacco samples were subjected to sequential membrane ultrafiltration using precise molecular weight cutoffs of 0.2 µm, 300 kDa, 50 kDa, and 1 kDa. The resulting retentates and filtrates, alongside their combusted smoke particulates generated *via* a smoking machine, were systematically characterized using UV-*vis* spectrophotometry, ^1^H NMR, and nontargeted analysis based on GC-MS and LC-HRMS. By tracking chemical markers across these separation stages, this investigation aims to identify the specific compounds distinguishing each tobacco sample, map their mechanistic transfer from uncombusted extracts into mainstream smoke particulates, and elucidate the critical role of bulk macromolecules as physical matrices governing this thermal transition.

## 2 Materials and methods

### 2.1 Sample description

Two tobacco samples, i.e., Zimbabwe tobacco leaf (Loft2108) and Hehua tobacco dust, were provided by China Tobacco Hebei Industry Co., Ltd. Both samples investigated in this study were flue-cured and balanced to a water content of 12%. Specifically, the Zimbabwe sample consisted of single-origin flue-cured tobacco leaf (Batch No. Loft2108). This leaf served as a key raw material in the formulation of the “Hehua” (Lotus) cigarette series. It exhibits a distinctive cigar-like sensory profile characterized by caramelized sweetness, resinous aroma, bean-like and honey-like notes, green nutty nuances, and roasted nutty characteristics, alongside desirable smoke attributes in terms of strength, diffusiveness, and concentration. The Hehua tobacco dust consists of a proprietary blend of this Zimbabwe flue-cured leaf and several domestic flue-cured tobacco leaves in mainland China. This dust was collected from particulate residues generated during the manufacturing processes of Hehua cigarette brand, originating from fine tobacco particles produced during cutting, blending, and cigarette-forming operations.

### 2.2 Sample preparation

Tobacco extracts were prepared using ultrasonic-assisted extraction. Both the Zimbabwe tobacco leaf and Hehua tobacco dust were separately weighed (50.0 g each), ground, and extracted with 70% (v/v) ethanol under ultrasonic treatment at room temperature for 45 min. The mixtures were filtered to separate the liquid phase from the solid residues. The residues were subjected to a second extraction under identical conditions, and the filtrates were combined. The combined extracts were concentrated under reduced pressure at 45 °C using rotary evaporation to remove solvents, yielding tobacco total extract.

The total extract was redissolved in 30% (v/v) ethanol and centrifuged at 10, 823 × g for 10 min at 4 °C. The supernatant was filtered and subsequently passed through a 0.2 μm membrane filter. Afterwards, the membrane was rinsed with 30% ethanol (1 L) to ensure complete transfer of retained compounds, and the combined filtrates were further concentrated. Sequential ultrafiltration was then performed using membranes with molecular weight cut-offs (MWCOs) of 300 kDa, 50 kDa, and 1 kDa, respectively. In brief, membrane separation was performed using a cross-flow ultrafiltration/nanofiltration unit (Chengda, Chengdu, China) equipped with online turbidity and conductivity monitoring, alongside an *in situ* cleaning system. The operating pressure was maintained between 0.1 and 0.3 MPa, with a cross-flow velocity of 1.0 to 1.5 m/s. The pH of the feed solution was adjusted to 6.0–7.0, and the concentration factor was maintained at 5–10. The cascade membrane filtration employed spiral-wound polyethersulfone (PES) membrane modules: a 300 kDa membrane (LX type, Synder Filtration, Suzhou, China), a 50 kDa membrane (MQ type, Synder Filtration, Suzhou, China), and a 1 kDa membrane (Hydranautics, Shanghai, China). To ensure optimal filtration efficiency and experimental reproducibility, all membrane modules underwent rigorous pretreatment prior to use. At each stage, both permeate and retentate fractions were collected and concentrated, yielding 300 kDa, 50 kDa, and 1 kDa permeate and retentate fractions.

For the chemical analysis on particulates in mainstream smoke of the tobacco extracts and their membrane-separated fractions, 0.3 g of each sample was diluted with equal volume of propylene glycol, followed by ultrasonication to ensure complete dissolution. Subsequently, 5 μL of each solution was loaded onto blank cigarettes using a microsyringe. The cigarettes were conditioned at 22 ± 2 °C and 60 ± 2% relative humidity for 48 h prior to smoking. The combustive smoking regime was executed strictly following the ISO 2012:3308 protocol: a 35 mL puff volume drawn over a 2.0 s puff duration, a 60 s interval between consecutive puffs, and unblocked filter ventilation. Mainstream smoke was collected using 44 mm Cambridge filter pads, with each pad corresponding to five cigarettes. A total of 20 cigarettes were used per sample. After smoking, filter pads and wiping cotton were cut into small pieces, combined, and extracted with 20 g of absolute ethanol under ultrasonication at 40 °C for 30 min. The extract was centrifuged, and the supernatant was collected and transferred to HPLC vials for further analysis.

### 2.3 UV-*vis* analysis

UV–*vis* spectra of membrane-separated and filter pad samples were acquired using a UV–*vis* spectrophotometer (Cary 5000, Agilent Technologies, Santa Clara, CA, USA). For each fraction, 10 μL of sample solution was diluted with 4.0 mL of the corresponding solvent and thoroughly mixed before analysis. Blank solvents were used as references for baseline correction. Measurements were conducted at room temperature (25 ± 1 °C) over a wavelength range of 200–800 nm, using a 1 nm sampling interval and a scan rate of 600 nm per minute to ensure sufficient spectral resolution.

### 2.4 ^1^H NMR analysis

^1^H NMR spectra were acquired using a Bruker Avance NEO 600 MHz nuclear magnetic resonance spectrometer (Bruker BioSpin GmbH, Rheinstetten, Germany). For the membrane-separated samples, approximately 5 mg (dry weight) of each fraction was freeze-dried overnight using an LGJ-12A freeze dryer (Beijing, China) and dissolved in 0.55 mL D_2_O. After vortex mixing for 1 min, the solutions were transferred into 5 mm NMR tubes for analysis. For filter pad samples, materials were first dried under a nitrogen stream and reconstituted in 0.55 mL D_2_O, followed by ultrasonication for 10 min. After standing at room temperature for 2 h, the supernatants were collected and transferred into 5 mm NMR tubes. All NMR experiments were performed at 298 K under a magnetic field strength of 14.1 T, with 32 scans acquired for each sample.

### 2.5 GC-MS analysis

GC-MS analysis was performed using an Agilent 8890 gas chromatograph coupled with an Agilent 5977C mass selective detector (Agilent Technologies, USA). Prior to analysis, samples were dissolved in propylene glycol and diluted 1000-fold with ethanol to reduce matrix effects and improve analytical compatibility. The concentration of megastigmatrienone in the stock solution was not less than 10% (w/w), and it was used as a reference compound to monitor instrumental stability and chromatographic performance. Chromatographic separation was achieved on an HP-5MS capillary column (30 m × 0.25 mm × 0.25 μm). Helium was used as the carrier gas under constant flow mode at a flow rate of 1.0 mL/min. The injector temperature was maintained at 250 °C, and splitless injection mode was applied. The injection volume was 1.0 μL. The oven temperature program was set as follows: the initial temperature was 50 °C with no holding time, increased to 250 °C at a rate of 5 °C/min, and held for 10 min. The mass spectrometer was operated in electron ionization (EI) mode at 70 eV. The ion source temperature was 230 °C, the transfer line temperature was 250 °C, and the quadrupole temperature was 150 °C. The solvent delay time was set to 5 min. Mass spectra were acquired in full scan mode (m/z 50–550). Compound identification was performed by processing Total Ion Current (TIC) data through the Agilent MassHunter software suite. Experimental mass spectra were matched against the NIST20 mass spectral library. To ensure annotation rigor, specific identification criteria were established: only library hits with a spectral match factor greater than 70% were accepted. Furthermore, these spectral annotations were corroborated using experimental Retention Indices (RI). The detailed identification metrics, including the match scores and RI values for all annotated compounds, are provided in **Supplementary Data Sheet 1**. Quantification was performed using the peak area normalization method.

### 2.6 LC-HRMS analysis

LC-HRMS analysis was carried out using a Waters UPLC system coupled with a Sciex 5600 TOF MS instrument. Chromatographic separation was conducted using both reversed-phase liquid chromatography (RPLC) and hydrophilic interaction liquid chromatography (HILIC) to enhance metabolite coverage across a broad polarity range. RPLC separation was performed on a Waters ACQUITY UPLC HSS T3 column (130 Å, 1.7 µm, 2.1 × 100 mm), whereas HILIC separation was carried out on a Waters ACQUITY UPLC BEH Amide column (130 Å, 1.7 µm, 2.1 × 100 mm). Both chromatographic modes employed a binary mobile phase system consisting of water and acetonitrile, with 0.1% formic acid added to both phases. The flow rate was set at 0.3 mL/min, and the column temperature was maintained at 40 °C. Quality control (QC) samples were prepared by pooling equal volumes of all samples and analyzed using a standardized nine-gradient elution program (Wang et al., 2025). In brief, both the RPLC and HILIC nine-gradient systems feature an initial 1.5-minute isocratic hold and a final column wash from 24 to 27 minutes. For RPLC, the gradient begins at 99% water (Phase B). Gradients 1–5 decrease to 10% water at progressively slower rates, while Gradients 5–9 utilize a fixed 23-minute gradient time to reach progressively shallower target concentrations (10%, 30%, 50%, 70%, and 90%), ending with a 2% water wash. For HILIC, the gradient starts at 1% Phase B. Gradients 1–5 increase Phase B to 45% at progressively slower rates, reaching the target at 5, 10, 15, 20, and 23 minutes, whereas Gradients 5–9 use a fixed 23-minute gradient time to reach progressively lower final concentrations (45%, 35%, 25%, 15%, and 5%), ending with a 49% Phase B wash.

MS scan was performed using a data-independent acquisition (DIA) strategy in both positive and negative ionization modes (Wang R.-Q. et al., 2023). TOF MS full-scan data were acquired over an m/z range of 70–1200 Da with an accumulation time of 150 ms. The DIA method employed 12 variable-width SWATH windows, with an MS/MS scan time of 50 ms for each window. The declustering potential (DP), collision energy (CE), and collision energy spread (CES) were set to 80 V, 35 V, and 15 V, respectively. Comprehensive data processing and nontargeted metabolite annotation were conducted using the integrated chromatographic retention behavior–fragmentation chain characterization (CRB-FCC) workflow (Wang et al., 2021; Wang et al., 2025). Based on the nontargeted ion feature list extracted from the QC dataset using the CRB algorithm, targeted searches were performed for corresponding nontargeted ion peaks in the LC-MS/MS data of each designated sample. For each extracted feature, the FCC algorithm was subsequently applied to analyze MS/MS fragmentation patterns through fragment ion and neutral loss chain matching, thereby enabling molecular formula assignment and structural annotation (Wang et al., 2025). Subsequently, level 2 annotations of nontargeted analysis ion features were attained by matching their experimental mass spectra with MassBank of North America (MoNA) (https://mona.fiehnlab.ucdavis.edu/), with mass error (ME) less than 10 ppm and dot product (DP) value beyond 0.4 (Schrimpe-Rutledge et al., 2016). Then, the peak area of each ion feature was integrated across the two investigated tobacco samples. The ion features showing significant group variances, i.e., fold change > 2, p-value < 0.05, and VIP score > 1 were recognized as markers differentiating the two tobacco samples.

## 3 Results and discussion

### 3.1 Spectral analysis of extracts and mainstream smoke particulates from two tobacco samples and their membrane fractions

**Figure 1:**
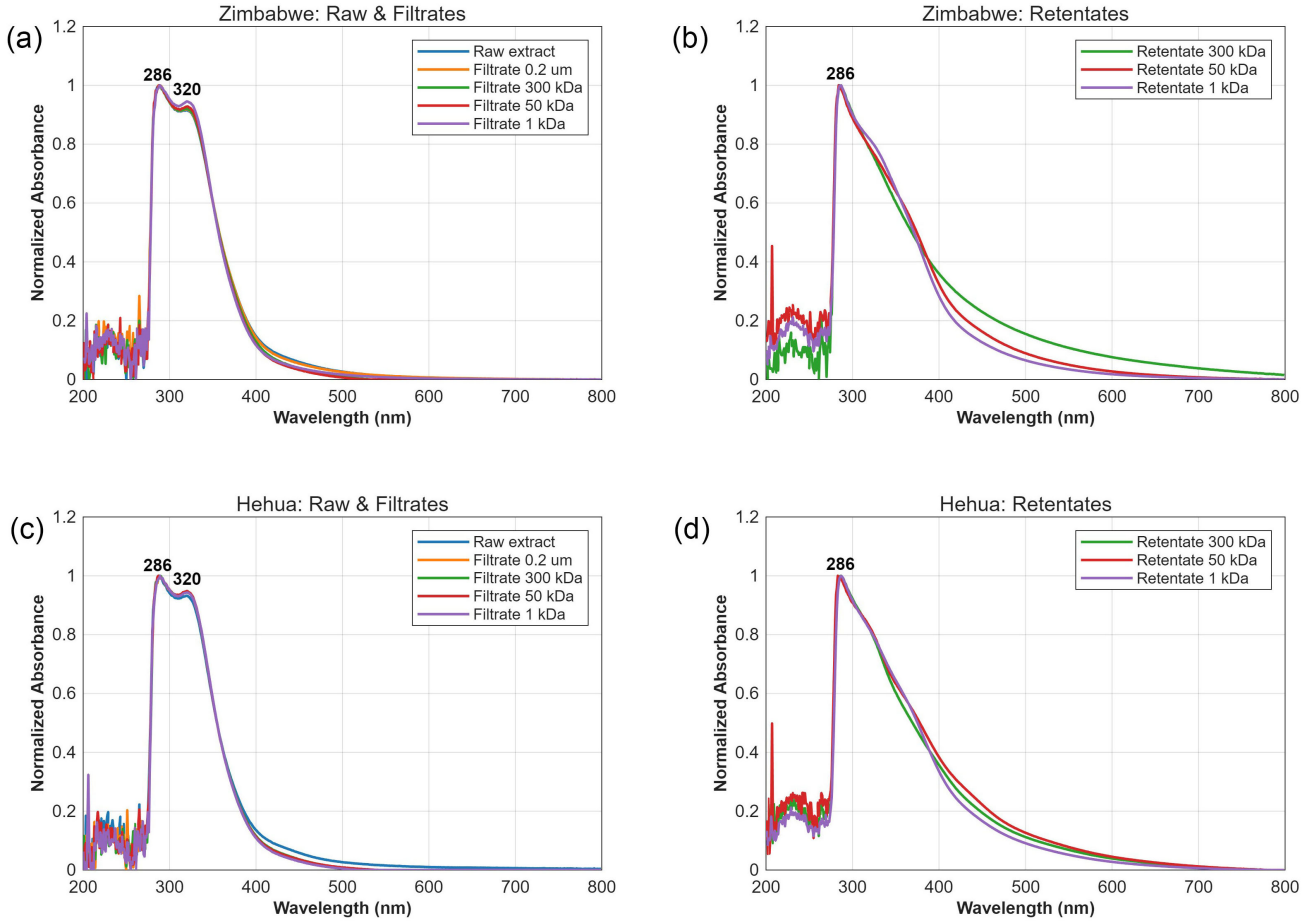
Comparative UV-*vis* spectral study of extracts from Zimbabwe tobacco leaf and Hehua tobacco dust. UV-*vis* spectra are shown for **(a)** the Zimbabwe tobacco raw extract and its corresponding membrane filtrates, **(b)** the retentates isolated from the Zimbabwe raw extract, **(c)** the Hehua tobacco dust raw extract and its corresponding membrane filtrates, and **(d)** the retentates isolated from the raw extract of Hehua tobacco dust.

Both extracts from Zimbabwe tobacco leaf and Hehua tobacco dust exhibited a prominent absorption peak at 286 nm (**Figure 1**). According to previous literature on herbal plant extract analysis, this specific wavelength is characteristic of phenolic compounds (Spiridon et al., 2011). Additionally, a shoulder peak was observed at 320 nm, which is characteristic of conjugated cinnamoyl structures such as chlorogenic acid (Fujioka and Shibamoto, 2008). Following cascade membrane fractionations, the filtrates of both tobacco extracts retained this dual-peak spectral signature. However, the retentates isolated by the 300 kDa molecular weight cut-off (MWCO) membrane lost the 320 nm shoulder peak, with only the 286 nm peak retained. This spectral shift confirms that conjugated structures of lower molecular weight, such as free chlorogenic acid, successfully passed into the filtrate. Conversely, the retentate consisted primarily of high molecular weight species, including polymeric phenols and complex matrices, which retain chromophores that absorb at 286 nm.

Despite the stark contrast in their sensory perceptions, the UV spectra of both tobacco extracts were nearly identical. Furthermore, the retentates also exhibited highly similar spectroscopic features. This striking spectral parity indicates that the bulk macromolecular compositions of both tobacco varieties share highly conserved structural features. Consequently, the distinct aroma compounds responsible for their intense sensory perceptions are likely minor components relative to the highly abundant pool of phenols and chlorogenic acids. Because these bulk phenolic compounds present prominent, overlapping absorption bands that dominate the UV spectra, the distinct trace aroma precursors remain optically masked. This highlights that while bulk macromolecular structures are conserved between the two varieties, trace variations within these retained macromolecular fractions are the critical drivers of their divergent sensory profiles.

**Figure 2:**
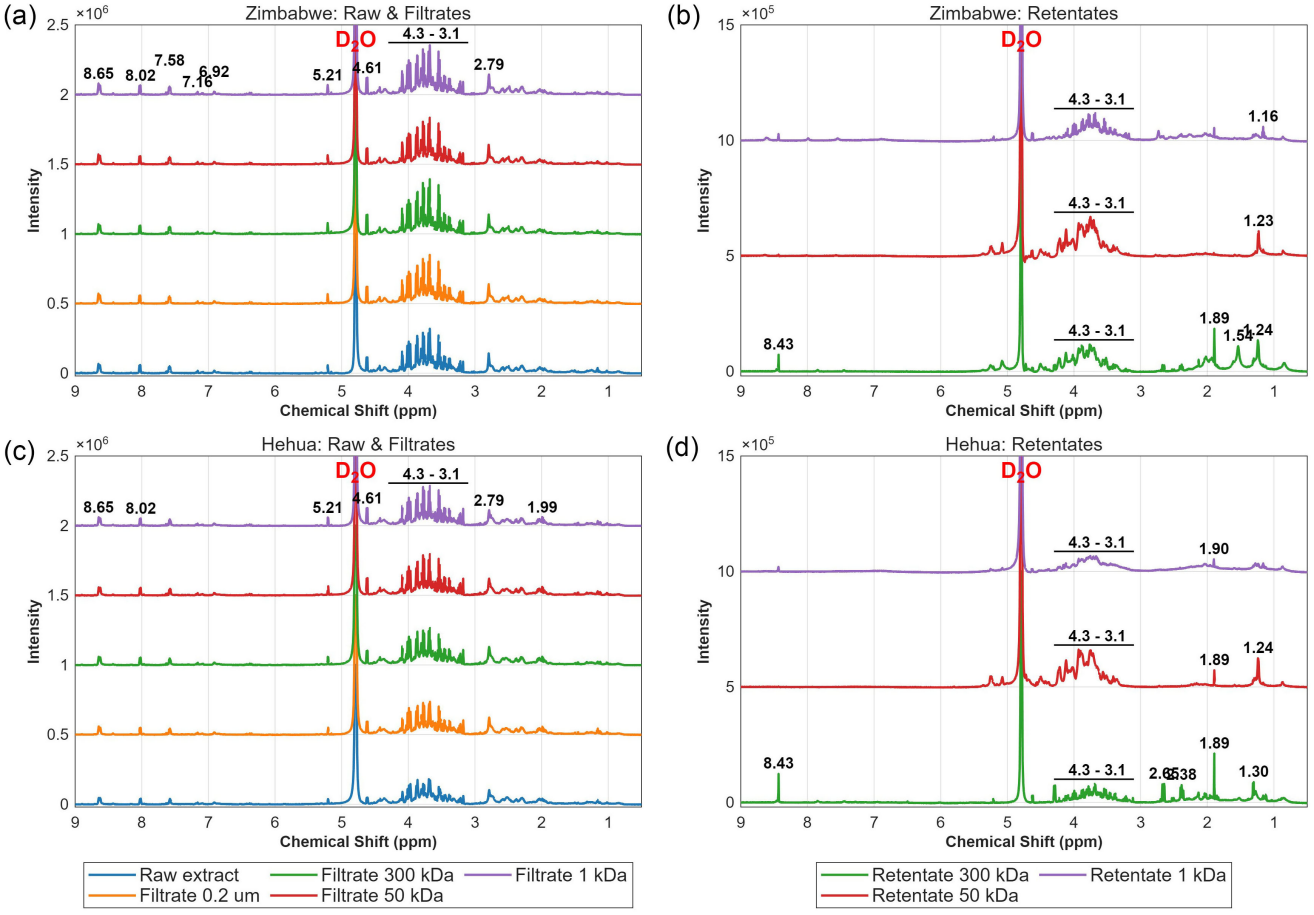
Comparative ^1^H NMR characterizations of Zimbabwe and Hehua tobacco samples. ^1^H NMR spectra are shown for **(a)** the Zimbabwe tobacco raw extract and its corresponding membrane filtrates, **(b)** the retentates isolated from the Zimbabwe raw extract, **(c)** the Hehua tobacco raw extract and its corresponding membrane filtrates, and **(d)** the retentates isolated from the Hehua raw extract.

Consistent with the UV-*vis* spectral results, ^1^H NMR spectra demonstrated striking parity between the unfractionated Zimbabwe and Hehua tobacco extracts, as well as across their respective membrane fractions (**Figure 2**). This broad spectral conservation further confirms that the bulk macromolecular and small-molecule profiles of both tobacco samples are fundamentally analogous. In the raw extracts and filtrates, distinct peaks were observed in the highly deshielded aromatic region at 8.65, 8.02, 7.58, 7.16 and 6.92 ppm. These resonances are characteristic of the pyridine ring protons of nicotine and structurally related nitrogenous alkaloids (Duell et al., 2018). Furthermore, prominent signals identified at 5.21 and 4.61 ppm correspond to the anomeric protons of reducing carbohydrates, specifically the α- and β-anomers of glucose (Periyannan et al., 2015). In the aliphatic region of the raw extracts and filtrates, a concentrated cluster of sharp, well-resolved signals is prominently observed at 1.99 and 2.79 ppm. These peaks specifically characterize the aliphatic methylene protons (-CH_2_-) situated on the carbon backbone immediately adjacent to the carboxyl groups, primarily representing the highly abundant malic and citric acids in tobacco (Rodrigues et al., 2010; Geng et al., 2023). Notably, these characteristic peaks do not represent the actual acidic protons (-COOH) of organic acids, as those rapidly exchange with the D_2_O solvent and remain unobservable. Instead, the highly electronegative carboxylic moiety exerts an electron-withdrawing effect that decreases the local magnetic shielding of the adjacent methylene protons. This deshielding shifts their resonance significantly downfield from the typical bulk aliphatic envelope (0.9–1.5 ppm) and into the distinctive 1.99–2.79 ppm window. The sharp and well-resolved peaks located in 4.61-8.65 ppm and 1.99-2.79 ppm ranges were consistently observed in both the unfractionated extracts and filtrates of the two investigated tobacco samples, but were completely absent in the membrane-isolated retentates. This striking contrast demonstrates that the identified constituents, such as alkaloids, simple sugars, and organic acids, are of sufficiently low molecular weight (i.e., < 1 kDa) to freely permeate the membranes. Due to their marginal UV absorbance, these compounds remain undetected in the UV spectra of both the crude tobacco extract and the filtrates.

In the region between 3.1 and 4.3 ppm, a broad hump may indicate compounds with carbohydrate skeletons (Brown et al., 2018). In both the raw extracts and the filtrates, this region exhibited numerous sharp, well-defined peaks indicative of small and freely tumbling saccharides with highly resolved fine structures. Conversely, in the retentates, this region manifested as a featureless and broad band. This spectral broadening is a classic NMR signature of macromolecules (Wishart, 2008). The restricted mobility and varied microenvironments of polymeric carbohydrates and complex matrices cause rapid transverse relaxation, resulting in unresolved resonance bands (Jones and Mulloy, 1993). The presence of carbohydrates in both the filtrate and retentate demonstrates a broad molecular weight distribution, encompassing both low-molecular-weight simple sugars and high-molecular-weight polysaccharides. Furthermore, the strong dominance of carbohydrate proton signals in the retentate indicates that these polysaccharides constitute the primary macromolecular component of the sample.

Further characterization of the high-molecular-weight retentates revealed prominent signals in the highly shielded aliphatic region, specifically at 1.24 and 1.89 ppm for both tobaccos, alongside a 1.54 ppm peak unique to the Zimbabwe extract. The intense peak at 1.24 ppm is attributed to protons on aliphatic chains, such as cuticular waxes or lipid-bound polymers. These aliphatic signatures are prominent in the retentate isolated by 300 kDa membrane, but subsequently decreased in the 50 kDa and 1 kDa isolates. The result indicates the size-based exclusion of these high-molecular-weight, non-polar complexes.

Ultimately, because UV and ^1^H NMR confirmed that the bulk macromolecular matrices of the two samples are highly conserved, it became evident that their distinct sensory profiles must be driven by trace chemical divergences, necessitating the comprehensive MS profiling detailed in the following sections.

### 3.2 Nontargeted analysis of mainstream smoke particulates from tobacco extracts and their membrane fractions by GC-MS

GC-MS was employed to compare the chemical compositions of mainstream smoke particulates generated from raw extracts of Zimbabwe tobacco and Hehua tobacco dust, as well as the mainstream smoke particulates from the membrane fractionated components of the two tobacco extracts. Because the evaluated samples were loaded onto the blank cigarettes at very low concentrations (i.e., ppm level), compounds derived specifically from the evaluated tobacco extracts were selected only if their signal intensities were at least three times higher than those observed in the blank tobacco mainstream smoke particulates. From this filtered dataset, 17 core compounds differentiating the two tobacco extracts were successfully identified (Data Sheet 1, Supporting Information).

Zimbabwe extract exhibited a significantly higher overall volatile abundance compared to the Hehua extract. The smoke particulates derived from cigarettes treated with Zimbabwe raw extract yielded roughly double the total signal intensity at approximately 1.83 × 10^6^ units, compared to the 9.31 × 10^5^ units observed in the Hehua smoke particulates. Furthermore, analyzing the differential abundance revealed a clear quantitative dominance in the Zimbabwe sample, with 10 major compounds showing significantly higher abundance in Zimbabwe, whereas 7 major compounds dominated in Hehua. This quantitative discrepancy suggests that the Zimbabwe variety provides a higher concentration of volatile precursors, which likely contributes to the intensity of its aromatic profile compared to the relatively subtler Hehua tobacco dust.

**Figure 3:**
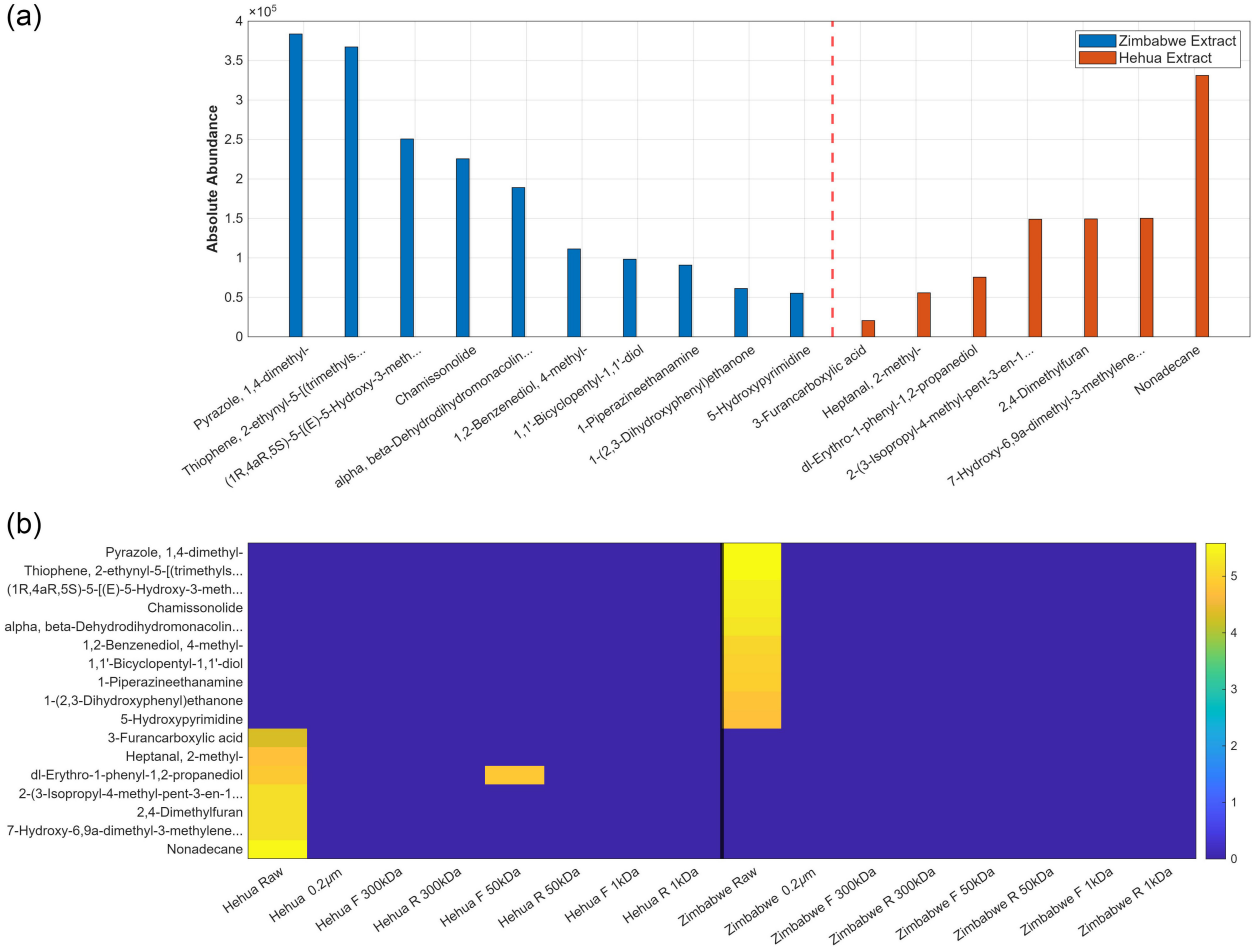
Comparative GC-MS profiling of characteristic compounds in the mainstream smoke particulates generated from the combusted Zimbabwe and Hehua tobacco extracts and their membrane ultrafiltration fractions. **(a)** Absolute abundance of the 17 core compounds in the raw extracts, sorted by their abundance difference between the two tobacco types. The red dashed line separates Zimbabwe-dominant and Hehua-dominant compounds. **(b)** log10-transformed heatmap illustrating compound distribution across all separation stages [raw, 0.2 μm pre-filtered, and 300/50/1 kDa filtrates (F) and retentates (R)]. A black vertical line separates the Hehua and Zimbabwe sample suites.

The comparative chemical signature differentiating the two tobaccos is driven by highly specific constituent variations (**Figure 3a**). The compound exhibiting the highest positive concentration difference in Zimbabwe is 1, 4-dimethylpyrazole, followed by 2-ethynyl-5-[(trimethylsilyl)ethynyl]thiophene and various complex carbaldehydes. Pyrazoles are well documented in tobacco chemistry for their roles in contributing nutty, roasted, and toasted sensory attributes, which align with the characteristically bold profile of Zimbabwe-type leaf (Zhao et al., 2026). Simultaneously, the thiophene derivatives identified are key heterocyclic contributors to smoky roasted aromas (Parr et al., 2023). Conversely, the chemical profile of Hehua tobacco dust was characterized by higher relative concentrations of heavy alkanes such as nonadecane, as well as specific furans like 2, 4-dimethylfuran and 7-hydroxy-6, 9a-dimethyl-3-methylene-decahydro-azuleno[4, 5-b]furan-2, 9-dione. Furans are critical for delivering sweet, caramel, and nutty nuances (Genovese et al., 2025). Nonetheless, high concentrations of long-chain alkanes like nonadecane often contribute to an undesirable waxy or oily mouthfeel (Wang Z. et al., 2023). These variations in nitrogen-containing heterocycles and sweet-toned oxygenates likely dictate the fundamental principles of the sensory differences between the Zimbabwe and Hehua tobacco samples.

Before membrane separation, the liquid extracts of the two highly contrasting tobacco types, Hehua and Zimbabwe, display distinct compositional differences. However, when these extracts are subjected to membrane ultrafiltration and the resulting filtrates and retentates are subsequently applied to blank cigarettes for combustion, an unexpected phenomenon occurs. GC-MS analysis of the smoke particulates from those membrane fractions reveals a complete depletion of their defining characteristic compounds, such as the Zimbabwe-dominant pyrazoles and Hehua-dominant furans (**Figure 3b**). This indicates that during the ultrafiltration of the liquid extracts, the key chemical precursors responsible for generating these unique volatile signatures in the smoke are either irreversibly adsorbed onto the polymeric membrane material or lost to process-induced volatilization (Guo et al., 2019; Zhang et al., 2023). Consequently, GC-MS analysis is unable to elucidate the true chemical differences between the membrane-separated samples. Because GC-MS inherently focuses on volatile compounds, it targets a chemical fraction whose identifying markers have already been stripped away, entirely missing the non-volatile macromolecules that likely constitute the major components of the separated fractions. Furthermore, the volatile profiles captured from the smoke particulates are overly simplified and fail to reflect the complex compositional divergence of the extracts. Ultimately, because ultrafiltration acts as a stripping mechanism that permanently alters the liquid matrix, relying solely on the volatile signatures of the downstream smoke is inadequate for characterizing the true nature of the separated extract fractions. Because GC-MS volatile profiling is heavily compromised by these process-induced filtration losses, investigating the potential interactions between small molecules and macromolecular carriers required shifting our focus to the non-volatile precursor matrix *via* LC-HRMS.

### 3.3 Nontargeted analysis of tobacco extracts and their membrane ultrafiltration fractions by LC-HRMS

LC-HRMS-based nontargeted analysis was conducted to comprehensively identify distinguishing characteristics between the Zimbabwe and Hehua samples. Features were rigorously filtered based on their Variable Importance in Projection (VIP > 1.0) and univariate statistical significance (p < 0.05). Applying these strict thresholds to the dataset yields a total of 4, 152 characteristic ion features. The distribution reveals a distinct chemical asymmetry, with 3, 003 features specifically marking the Hehua tobacco dust and 1, 149 features characterizing the Zimbabwe tobacco leaf (Data Sheet 2, Supporting Information). Within this pool of highly significant markers, the annotation success varies dramatically. Molecular formulas were successfully assigned to the vast majority of the chemical markers: 2, 727 (90.8%) for Hehua and 1, 088 (94.7%) for Zimbabwe. However, the success rate for structural annotation was much lower, with tentative structures assigned to only 340 (11.3%) of the Hehua markers and 182 (15.8%) of the Zimbabwe markers. The structural annotation of specialized secondary metabolites endemic to tobacco is inherently limited by the profound lack of natural product records in existing spectral databases (Nakabayashi and Saito, 2013). Nevertheless, the implementation of *de novo* elucidation workflows, i.e., the Fragment Chain Characterization algorithm, provides a robust alternative, facilitating the broad formula assignment of these previously uncharacterized markers (Wang et al., 2025). According to the standardized confidence levels for HRMS (Schymanski et al., 2014), the tentative structures achieved through conventional database matching can be classified as Level 2a annotations (probable structure by library spectrum match), whereas the structural assignments deduced by the FCC algorithm utilizing diagnostic fragment ions are categorized as Level 2b annotations (probable structure by diagnostic evidence).

**Figure 4:**
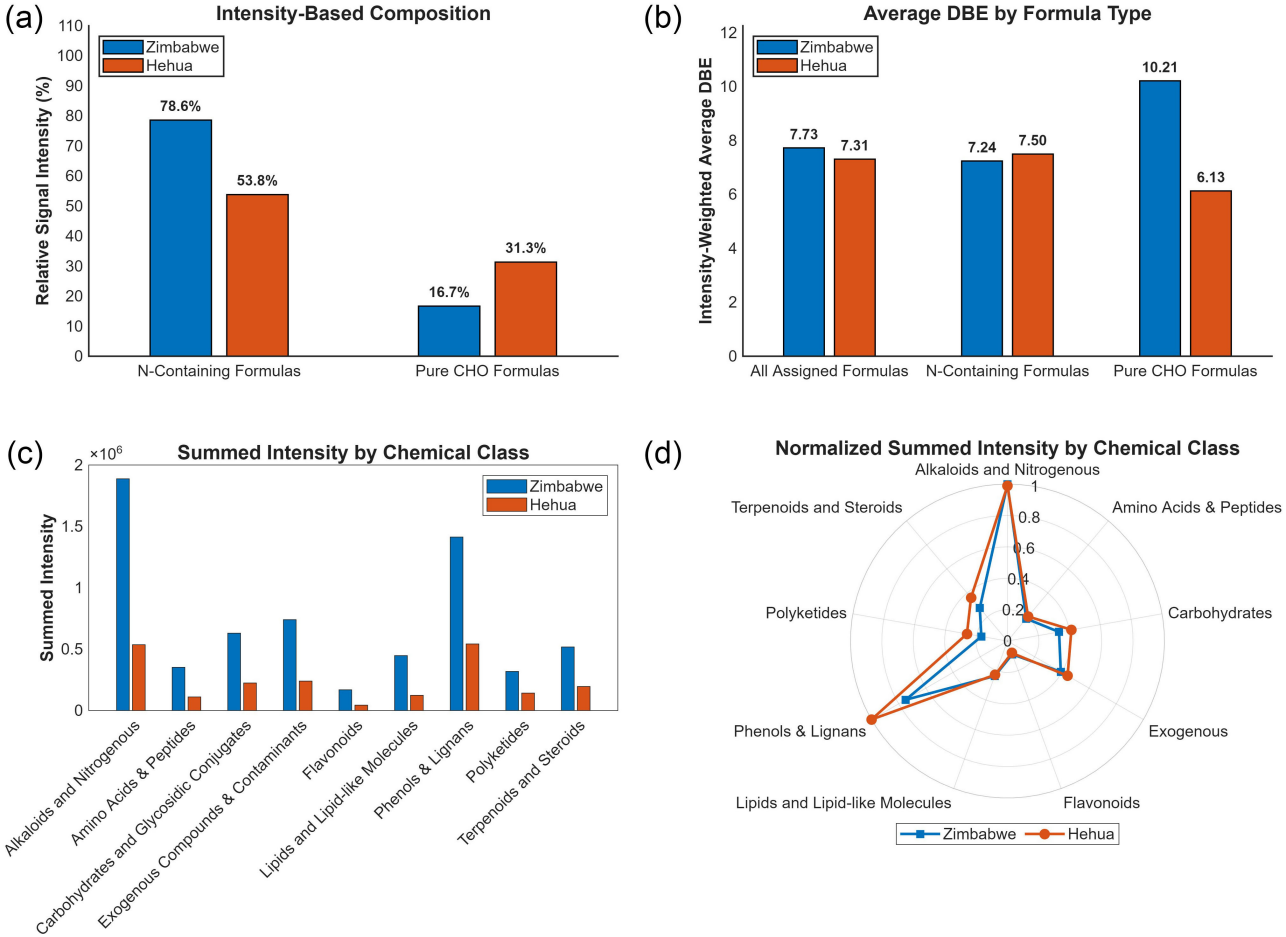
Comparative structural and compositional characterization of the discriminative markers identified in the extracts of Zimbabwe tobacco leaf and Hehua tobacco dust by nontargeted analysis based on LC-HRMS. **(a)** Relative signal intensity distribution of the assigned elemental formulas, highlighting the contrast between nitrogen containing compounds and pure carbon, hydrogen, and oxygen constituents; **(b)** Intensity-weighted average double bond equivalents calculated across the different elemental classes, revealing the highly saturated and aliphatic profile of the Hehua extract; **(c)** Absolute signal intensities of the structurally annotated compounds categorized into nine fundamental chemical classes; **(d)** Relative abundance profiles of the annotated chemical classes, illustrating the altered proportional balance between alkaloids, nitrogenous derivatives, phenols, and lignans across the two tobacco samples.

The analysis revealed that the discriminative markers for both tobacco samples were predominantly composed of complex, heteroatom-containing macromolecules. By formula count, nitrogen-containing formulas were nearly ubiquitous, accounting for 79.3% (2, 163) of the Hehua markers and 75.9% (826) of the Zimbabwe markers. Furthermore, almost all assigned formulas (>97%) contained oxygen. The near-ubiquitous co-occurrence of oxygen within these nitrogenous markers (>97%) reflects the intense oxidative metabolism, glycosylation, and Maillard-driven sugar-amino acid conjugations characteristic of cured tobacco matrices. When evaluating relative signal intensity, the structural variance of Zimbabwe tobacco was overwhelmingly driven by nitrogenous compounds. Nitrogen-containing markers accounted for 78.6% of the relative intensity in the Zimbabwe extract, vastly overpowering its purely oxygenated (CHO) counterparts, which made up only 16.7%. As such, the intensity of nitrogen-containing markers is 3.7-fold higher than that pure CHO markers in Zimbabwe extract. This massive reserve of nitrogen-containing markers in the liquid matrix serves as the direct thermal precursor pool for the volatile pyrazoles and N-heterocycles that uniquely characterized the Zimbabwe smoke particulates in the GC-MS analysis. Conversely, the intensity of pure CHO markers in Hehua comprised 31.3% of all the markers, in comparison to 53.8% nitrogen-containing counterparts. This high abundance of complex oxygenated precursors directly explains the pronounced generation of furans and sweet-toned oxygenated volatiles uniquely observed through the GC-MS analysis of Hehua smoke particulates (**Figure 4a**).

The intensity-weighted average double bond equivalent (*i*-DBE) reveals distinct structural profiles between the two samples. Across all assigned formulas, the Zimbabwe extract exhibits a slightly higher overall average DBE (7.06) compared to Hehua (6.79). When separated by chemical class, the nitrogen-containing formulas show a slightly higher average unsaturation in Hehua (7.50) than in Zimbabwe (7.24). However, the most striking divergence appears in the pure CHO formulas, where the Zimbabwe sample has a substantially higher average DBE (10.21) than Hehua (6.13). The results suggest that the core nitrogen-containing chemistry is relatively conserved between the two varieties. Instead, the primary difference between the samples is driven entirely by the pure CHO compounds. In Hehua extract, the pure CHO markers exhibited much lower *i*-DBE compared to Zimbabwe extract, indicating a significantly higher content of saturated, aliphatic structures, such as lipids, in the Hehua sample. This high aliphatic content strongly aligns with the GC-MS results, confirming the distinct, lipid-rich profile of the Hehua tobacco leaves compared to the more aromatic CHO profile of the Zimbabwe tobacco extract (**Figure 4b**).

To systematically evaluate the structural composition of the metabolome, the successfully annotated compounds were categorized into nine chemical classes: alkaloids and nitrogenous derivatives, amino acids and peptides, carbohydrates and glycosidic conjugates, flavonoids, lipids and lipid-like molecules, phenols and lignans, polyketides, terpenoids and steroids, as well as exogenous compounds and contaminants (**Figure 4c**). A comparative analysis of these classes reveals that the Zimbabwe extract exhibited substantially higher absolute intensities across all categories, yielding an overall signal approximately 3-fold higher than that of Hehua. Despite this significant quantitative gap, the overall pattern of intensity distribution remained largely conserved between the two tobacco samples. In both extracts, alkaloids and nitrogenous derivatives alongside phenols and lignans formed the dominant chemical backbone. Such non-volatile constituents were found as fundamental chemical pool shaping the sensory perceptions of tobacco (Geng et al., 2023).

Although the global chemical profiles are largely conserved, evaluating the relative abundances across chemical classes reveals pronounced compositional variations. Zimbabwe presents a traditional alkaloid-heavy profile, whereas Hehua demonstrates an altered chemical balance, featuring a near 1:1 relative abundance of phenols and lignans to alkaloids and nitrogenous derivatives (**Figure 4d**). Furthermore, Hehua exhibits slightly higher relative proportions of polyketides, terpenoids, and carbohydrates. While elevated carbohydrates and terpenoids can introduce desirable aromatic complexity, the atypically high proportion of phenols and lignans relative to alkaloids in Hehua suggests a smoke profile that may lean towards increased astringency and heaviness (Dai and Mumper, 2010; Schieber and Wüst, 2020; Fan et al., 2023). This altered ratio likely complicates the sensory perception of Hehua tobacco dust, potentially manifesting as an unfavorable or overly complex flavor profile upon combustion. Notably, the relative intensities of compounds successfully annotated as lipid and lipid-like molecules *via* standard database matching appear similar between the two tobacco samples. However, this superficial parity likely represents a significant underestimation of the actual lipid-rich nature of the Hehua matrix. The structural annotation of complex aliphatic networks, including endogenous plant lipids and cuticular waxes, is inherently bottlenecked by the limited coverage of empirical spectral libraries. Lipids exhibit massive combinatorial diversity, which far exceeds the capacity of conventional databases that are predominantly curated for primary metabolites and common natural products (Kind et al., 2013). Consequently, a substantial fraction of the Hehua lipidome remains structurally unannotated, reinforcing the necessity of relying on bulk formula-level metrics to accurately reflect its saturated composition.

### 3.4 Tracking of chemical markers in mainstream smoke particulates generated from tobacco extracts and their membrane ultrafiltration fractions

**Figure 5:**
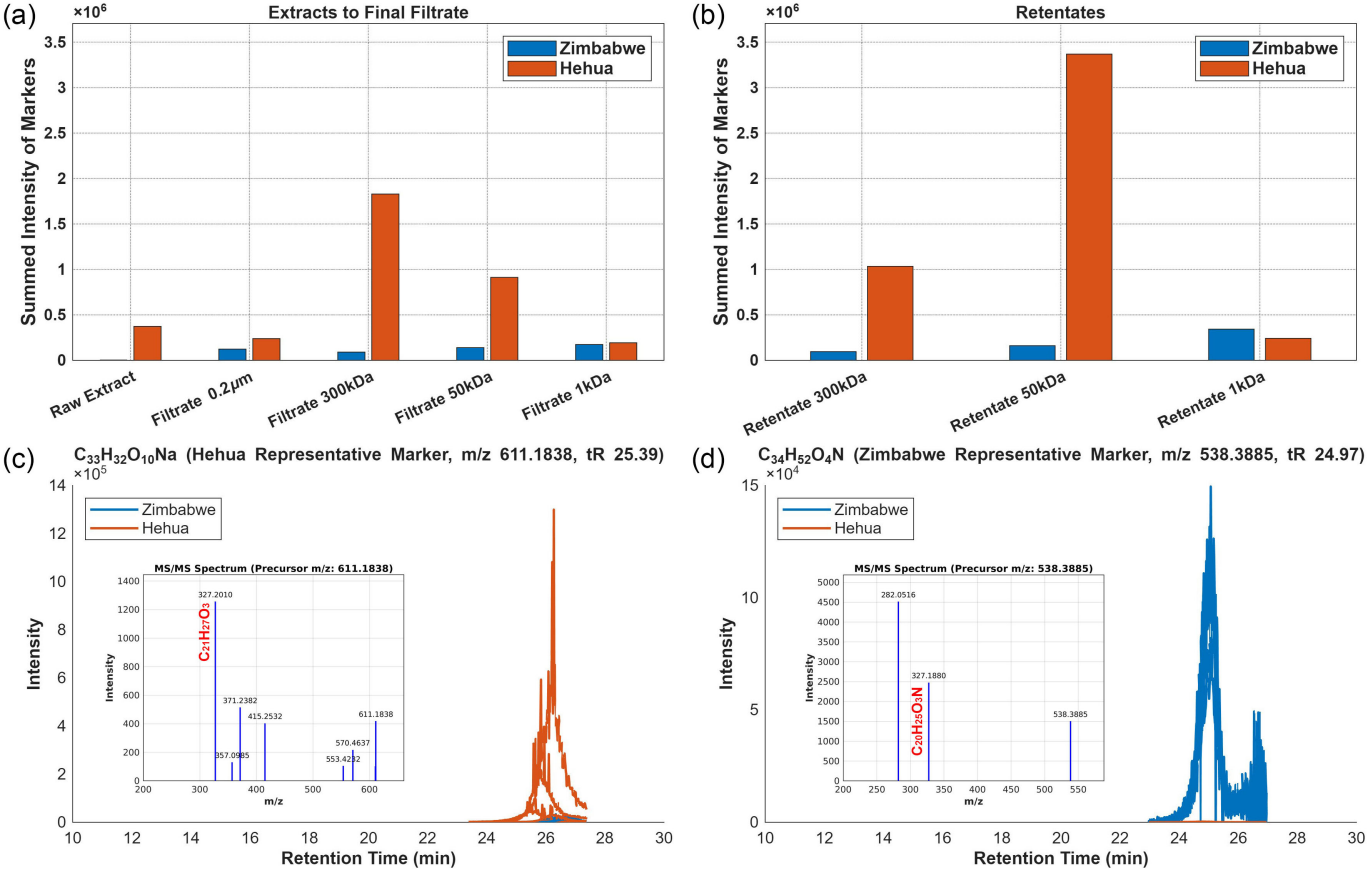
Tracking of chemical markers *via* LC-HRMS in smoke particulates derived from extracts of Zimbabwe tobacco leaf and Hehua tobacco dust and the cascade membrane fractions. **(a)** Summed intensity trends of markers in the smoke particulates of filtrate during the filtration sequence, demonstrating the systematic alleviation of matrix effects and ion suppression. **(b)** Summed intensity trends in the smoke particulates of corresponding retentates, illustrating the varying association between isolated macromolecules and free small molecules during filtration. **(c, d)** Extracted ion chromatograms of the most representative markers in the raw extracts and smoke particulates for Zimbabwe and Hehua tobacco samples, detected in RPLC positive ion mode. The MS/MS spectra and diagnostic ions of both markers are shown as insets, with structural formulas assigned using the FCC algorithm.

LC-HRMS analysis of the mainstream smoke particulates from the fractionated tobacco extracts successfully tracked 100 (8.7%) and 622 (20.7%) chemical markers for Zimbabwe and Hehua tobacco samples across the cascade membrane filtration sequence (Data Sheet 3, Supporting Information). The substantially lower tracking ratio of the Zimbabwe markers is likely attributable to their highly aromatic, nitrogen-rich structures. These characteristics promote strong intermolecular interactions with the polymeric membrane, leading to irreversible retention (Yamamura et al., 2007). For the Zimbabwe tobacco, the summed intensity of tracked markers in smoke particulates of the raw extract was exceptionally low (2.76 × 10^3^), but exhibited a steady, progressive increase during cascade filtration (**Figure 5a**). In contrast, the extract of Hehua tobacco dust demonstrated a more variable trend. While the initial marker intensity for its raw extract was also relatively low (3.73 × 10^5^), it surged to 1.83 × 10^6^ following filtration through a 300 kDa membrane, before gradually declining to 1.93 × 10^5^ in subsequent steps. The observed phenomenon highlights that for the LC-HRMS analysis of particulate-phase flavors, the influence of matrix effects is prominent. In both the Zimbabwe and Hehua tobacco samples, the initial intensities of markers in the smoke particulates of unfractionated raw extracts are significantly depressed. Following the cascade membrane filtration sequence, a critical clean-up mechanism systematically alleviates the ion suppression effects.

Analysis of the retentate fractions provides complementary insights into the partitioning of chemical precursors (**Figure 5b**). For the Zimbabwe tobacco, the summed intensity of the chemical markers progressively increased across the retentates attained through sequential membrane stages, climbing from 9.58 × 10^4^ to a peak of 3.42 × 10^5^. In stark contrast, the Hehua retentates mirrored the biphasic behavior. A massive surge in its markers intensities was observed from 1.03 × 10^6^ to 3.36 × 10^6^ between the 300 kDa and 50 kDa membrane isolations. Subsequently, the intensity sharply declined to 2.41 × 10^5^ in the smoke particulates derived from the 1 kDa retentate. Compared to the smoke particulates generated from the filtrates, the unexpectedly high marker intensities in the smoke particulates of the retentates suggest an association between the isolated macromolecules and the free small-molecule markers in the aqueous solution. Thus, we hypothesize these macromolecules may co-partition with the small molecules. Specifically, the Hehua tobacco fractions exhibited a much stronger co-enrichment with their respective markers than the Zimbabwe fractions. Given that a large proportion of the Hehua markers are pure CHO compounds with relatively low aromaticity, it is possible that these compounds possess a stronger tendency to interact with carbohydrate macromolecules compared to the highly aromatic, nitrogen-rich markers of the Zimbabwe tobacco. Notably, the highest retention of Hehua chemical markers was observed within the fraction containing macromolecules in the 50 to 300 kDa range. The removal of these specific macromolecules likely contributes to the significant decline in marker intensity in the downstream filtrates, coupled with the pronounced enrichment observed in the 50 kDa retentate.

Previous studies investigating differential metabolites across tobacco phenotypes have predominantly relied on traditional mass spectral library matching, which are inherently confined to a narrow chemical space defined by database records (Zhao et al., 2023; Zou et al., 2023). In contrast, our method employs the FCC algorithm to assign molecular formulas based on characteristic fragmental ions, effectively exceeding the boundaries of existing database records. The most representative markers for the Zimbabwe and Hehua tobacco samples were identified in RPLC positive ion mode as the ion features at m/z 538.3885 (tR = 24.97 min) and m/z 611.1838 (tR = 25.39 min), respectively. Although exact structural elucidation *via* existing databases was unsuccessful, their molecular formulas assigned by FCC algorithm indicate that the Zimbabwe marker is a nitrogen-containing compound, whereas the Hehua marker consists exclusively of carbon, hydrogen, and oxygen (CHO) (**Figures 5c, d**).

While the results strongly suggest this macromolecular association, further orthogonal physicochemical investigations are required to definitively confirm and quantify the exact mechanisms of these interactions. Additionally, highly aromatic and nitrogen-rich precursors are susceptible to irreversible adsorption onto polymeric membranes during cascade ultrafiltration, potentially obscuring the complete tracking of specific volatiles. Future research should prioritize these biophysical validations and address these methodological constraints.

## 4 Conclusion

This study establishes a comprehensive analytical framework linking non-volatile precursors to the particulate-phase flavor compounds formed after combustion. While UV-*vis* and ^1^H NMR revealed highly conserved bulk macromolecular profiles of carbohydrates in the extracts of both Zimbabwe tobacco leaf and Hehua tobacco dust, nontargeted LC-HRMS demonstrated that trace chemical divergences drive their contrastive sensory perceptions. Zimbabwe tobacco features an aromatic, nitrogen-rich matrix that GC-MS and LC-HRMS analyses of the mainstream smoke particulates confirm directly fuels the generation of desirable pyrazoles and other N-heterocycles in smoke. Conversely, Hehua tobacco dust exhibits a more complex balance of phenols and alkaloids, alongside a pure CHO fraction that is significantly more aliphatic and lipid-rich than Zimbabwe tobacco. Crucially, tracking representative markers through cascade ultrafiltration provided deep mechanistic insights into combustion transformations. The data revealed that specific macromolecules, particularly the 50 to 300 kDa fraction in Hehua, exhibit pronounced co-enrichment with free small-molecule precursors, suggesting a strong association that facilitates their co-partitioning during the transfer of these precursors into mainstream smoke. Ultimately, elucidating these precursor-to-smoke relationships and the associated macromolecular matrix dynamics establishes a robust, objective chemical foundation for the digital quality evaluation and targeted formulation of substitute tobacco materials.

## Data Availability

The original contributions presented in the study are included in the article/**Supplementary Material**. Further inquiries can be directed to the corresponding authors.
